# A Method for Estimating the Polarimetric Scattering Matrix of Moving Target for Simultaneous Fully Polarimetric Radar

**DOI:** 10.3390/s18051418

**Published:** 2018-05-03

**Authors:** Fulai Wang, Chao Li, Chen Pang, Yongzhen Li, Xuesong Wang

**Affiliations:** College of Electronic Science, National University of Defense Technology (NUDT), Changsha 410073, China; liusong.yue@163.com (C.L.); pangchen@nudt.edu.cn (C.P.); liyongzhen@nudt.edu.cn (Y.L.); wxs_2017@163.com (X.W.)

**Keywords:** polarization scattering matrix, moving target, simultaneous polarimetric radar, non-uniform motion

## Abstract

To precisely obtain the polarimetric scattering matrix (PSM) of moving target, a measurement model for the simultaneous fully polarimetric radar is formulated. The calibration errors and isolation of the transmitted waveforms are considered. To address the decline in performance of the traditional PSM estimation methods when the target moves, a novel method with measurement selection is proposed. Numerical experiments are conducted to demonstrate and validate the superiority of the proposed method, especially for the PSM estimation of the target with non-uniform motion.

## 1. Introduction

Advancements in radar technology and theory have provided a better understanding of the polarimetric information contained in radar targets [1,2,3,4]. The polarimetric features, which can be described by a second order polarimetric scattering matrix (PSM) have been widely used in various fields, such as terrain observation, disaster surveillance and atmospheric remote sensing. To accurately obtain the PSM, two fully polarimetric measurement schemes, called the alternately transmitting and simultaneously receiving (ATSR) scheme and the simultaneously transmitting and simultaneously receiving (STSR) scheme, have been widely investigated since the 1980s [5,6,7]. The ATSR radar alternately transmits waveforms through horizontal (H) and vertical (V) polarizations while both polarizations are received simultaneously on reception. At least two pulses are required in this mode to obtain the four elements for the PSM. The ATSR in essence is a time-sharing polarimetric radar, hence, the target decorrelation may influence the measurements results. In contrast, for STSR radar, the two orthogonal polarization states are transmitted and received simultaneously. Thus, the PSM of the targets can be retrieved within one pulse recurrent time (PRT). In this case, the limitation caused due to the change of the transmitted polarization states in ATSR scheme can be overcome [8].

Estimation of the target scattering matrix is based on the measurements from the fully polarimetric radar. For the static target, its echoes are coherent, and the pulse-integration (PI) method can be used to estimate the target’s PSM. For moving targets, the Doppler velocity which refers to the radial velocity, and the PSM of the target are coupled. It is unclear whether the change of the radar-echo phase is caused by the target’s displacement or the PSM. Therefore, the true PSM of the moving target is usually difficult to obtain [9,10,11]. Fortunately, in real radar applications, the PSM of a slow-moving target, such as that for an unmanned aerial vehicle (UAV), is assumed to be deterministic over the observation duration [12]. Under this assumption, provided that the target Doppler velocity is known, the phase changes in the radar echoes due to the target motion can be compensated by the estimated velocity. After the compensation, the incoherent echoes become coherent, and the PSM of moving target can be estimated by the pulse-compensation (PC) method. Obviously, the estimated accuracy of the PC method is related to the accuracy of the estimated Doppler velocity. When the velocity cannot be determined exactly, the estimated PSM becomes inaccurate [13].

To obtain the precise PSM of a moving target, a method of measurement selection (MS) is proposed in this paper. Using the criterion based on the signal to noise ratio (SNR) of the integration echoes, partial measurements are selected to estimate the PSM of the target. After the selection, the influences of the target motion on the four polarization channels can be considered uniform, and the PSM with relative amplitude and phase can be estimated. The advantage of this method is that the MS does not require any prior information about the target velocity, and it can still be used even if the target exhibits non-uniform motion. The rest of this paper is organized as follows: Section 2 presents the problem formulation; in Section 3, three PSM estimation methods are introduced; the numerical simulations are provided in Section 4 to verify the performance of the proposed method, followed by the conclusions in Section 5.

Notation: In this paper, it is assumed that a lower-case letter (e.g., *a*) denotes a scalar; a boldface lowercase letter (e.g., a) denotes a vector; and a boldface uppercase letter (e.g., A) indicates a matrix. Additionally, $A^{T}$ and $A^{H}$ denote the transpose and the conjugate transpose of the matrix A, and the symbol $\left|\cdot \right|$ denotes the modulus of a complex number.

## 2. Signal Model for Moving Target in STSR Radar

**Figure 1:**
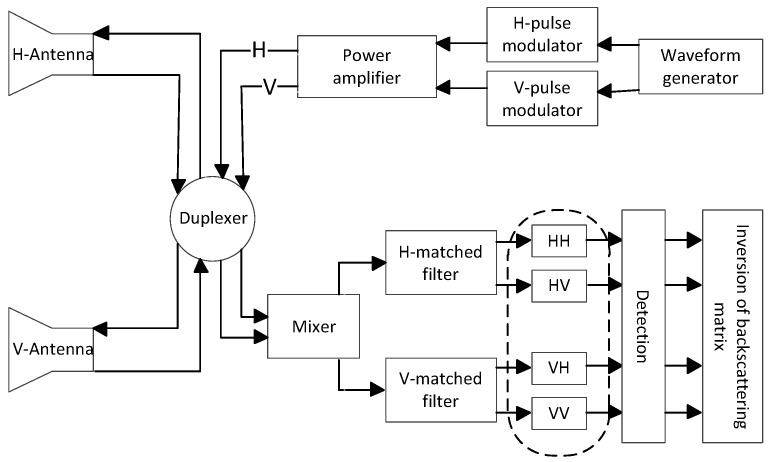
The signal processing flow chart of the STSR radar.

The simplified signal processing flow chart of the STSR radar is depicted in Figure 1 [8]. Suppose the STSR radar transmits a pair of opposite (up-going and down-going) slope of linear frequency-modulation (LFM) waveforms, which can be expressed as

(1) $$\left\{\begin{array}{c} s_{H}\left(t\right)=\mathrm{rect}\left(\frac{t}{T_{r}}\right)\exp\left(j2\pi f_{0}t+j\pi \gamma t^{2}\right)\\ s_{V}\left(t\right)=\mathrm{rect}\left(\frac{t}{T_{r}}\right)\exp\left(j2\pi f_{0}t-j\pi \gamma t^{2}\right) \end{array}\right.;t\in \left[-\frac{T_{r}}{2},\frac{T_{r}}{2}\right]$$

where

(2) $$\mathrm{rect}\left(\frac{t}{T_{r}}\right)=\left\{\begin{array}{c} 1,\;\left|t\right|\leq \frac{T_{r}}{2}\\ 0,\;\mathrm{else} \end{array}\right.$$

and $T_{r}$ is the pulse duration. $f_{0}$ is the carrier frequency, γ is the modulation slope, and the radar bandwidth is $B=\gamma T_{r}$. To facilitate the discussion, the transmitted waveforms can be given in vector form as

(3) $$s\left(t\right)={\left[s_{H}\left(t\right),s_{V}\left(t\right)\right]}^{T}$$

For a point target, the received signals are the time-delayed version of the transmitted signals. Thus, for the kth pulse, the received signal is [14]

(4) $$r_{k}\left(t\right)=\left[\begin{array}{c} r_{H,k}\left(t\right)\\ r_{V,k}\left(t\right) \end{array}\right]=R^{T}\cdot S_{k}\cdot T\cdot s\left(t-\frac{2d_{k}}{c}\right)+w_{k}\left(t\right);k=1,2,\ldots ,N$$

where *N* is the number of pulses in a coherent process interval (CPI), *c* is the speed of the light, $w_{k}\left(t\right)={\left[w_{H,k}\left(t\right),w_{V,k}\left(t\right)\right]}^{T}$ is the thermal noise of the receivers with variance $\sigma_{w}^{2}$, $d_{k}$ is the radial distance from the point target to the radar, and $S_{k}$ is the PSM of the target that can be described as

(5) $$S_{k}=\left[\begin{array}{c} S_{HH,k}\;S_{HV,k}\\ S_{VH,k}\;S_{VV,k} \end{array}\right]$$

where the corner marks HH, HV, VH and VV denote four polarization channels. R and T represent the effect of channels and antennas on the PSM during the reception and transmission, respectively. Here, the calibration errors of the STSR radar system, including the cross-polarization isolation of the antennas, the amplitude and phase difference of the channels, are considered. Suppose the same antennas and channels are used during the transmission and reception, then the R and T can be set as follows

(6) $$R=T=\left[\begin{array}{c} \;1\;\alpha_{HV}\exp\left(j\phi_{HV}\right)\\ \alpha_{VH}\exp\left(j\phi_{VH}\right)\;\alpha_{VV}\exp\left(j\phi_{VV}\right) \end{array}\right]$$

Processed by the mixer and matched filters shown in Figure 1, the high-resolution range profile (HRRP) can be obtained

(7) $$G_{k}\left(t\right)=R^{T}ST\left[\begin{array}{c} g_{HH,k}\left(t\right)\;\;g_{HV,k}\left(t\right)\\ g_{VH,k}\left(t\right)\;\;g_{VV,k}\left(t\right) \end{array}\right]+\left[\begin{array}{c} w_{HH,k}\left(t\right)\;\;w_{HV,k}\left(t\right)\\ w_{VH,k}\left(t\right)\;\;w_{VV,k}\left(t\right) \end{array}\right]$$

and

(8) $$g_{HH,k}\left(t\right)=A\cdot \sin c\left(\pi B\left(t-\frac{2d_{k}}{c}\right)\right)\cdot \exp\left(-j\frac{4\pi d_{k}}{\lambda }\right)$$

(9) $$g_{VV,k}\left(t\right)=A\cdot \sin c\left(\pi B\left(t-\frac{2d_{k}}{c}\right)\right)\cdot \exp\left(-j\frac{4\pi d_{k}}{\lambda }\right)$$

where $A=BT_{r}$ is the gain of the matched filtering (MF), λ is the wavelength, and $\sin c\left(\cdot \right)$ is the Sinc function. One thing should be pointed out is that, generally, the scattering matrix is related to the shape, geometrical structure, reflectivity and orientation of the target. Meanwhile, the PSM may fluctuate whether or not the target is in motion. Fortunately, this effect can be controlled by limiting the CPI of the radar system. If the CPI is short enough, the PSM of the target can be assumed to be same for different pulses. Thus, S is used to replace $S_{k}$ in Equation (7). With $t=2d_{k}/c$, Equations (8) and (9) can be written into

(10) $$g_{HH,k}=A\cdot \exp\left(-j\frac{4\pi d_{k}}{\lambda }\right)$$

(11) $$g_{VV,k}=A\cdot \exp\left(-j\frac{4\pi d_{k}}{\lambda }\right)$$

Additionally, in [15], the author has pointed out that the isolation of opposite slope of LFM waveforms, which is defined as

(12) $$I=\max_{\forall t}\;\left\{20\log_{10}\frac{\left|g_{HV,k}\left(t\right)\right|}{\max_{\forall t^{\prime }}\;\left|g_{HH,k}\left(t^{\prime }\right)\right|}\right\}$$

is related to the time-bandwidth product of the waveforms and an approximate equation is given as follows

(13) $$I\approx -10\log_{10}\left(BT_{r}\right)$$

Obviously, when the time-bandwidth product is large enough, such as $BT_{r}=10^{4}$, the *I* is equal to −40dB approximately, which means for arbitrary $t\in \left[-T_{r},T_{r}\right]$, the modulus of $g_{HV,k}\left(t\right)$ and $g_{VH,k}\left(t\right)$ are much less than that of $g_{HH,k}$ and $g_{VV,k}$. Therefore, when $t=2d_{k}/c$, Equation (7) can be further rewritten as

(14) $$G_{k}=\left[\begin{array}{c} G_{HH,k}\;G_{HV,k}\\ G_{VH,k}\;G_{VV,k} \end{array}\right]=R^{T}ST\left[\begin{array}{c} g_{HH,k}\;I^{\prime }\\ \;I^{\prime }\;g_{VV,k} \end{array}\right]+\left[\begin{array}{c} w_{HH,k}\;w_{HV,k}\\ w_{VH,k}\;w_{VV,k} \end{array}\right]$$

where $I^{\prime }=10^{I/20}$. If the second order small quantities are ignored, it comes

(15) $$\left\{\begin{array}{c} G_{HH,k}=g_{HH,k}\left(S_{HH}+\alpha_{VH}\exp\left(j\phi_{VH}\right)\left(S_{VH}+S_{HV}\right)\right)+I^{\prime }\cdot \alpha_{VV}S_{HV}\exp\left(j\phi_{VV}\right)+w_{HH,k}\\ G_{VH,k}=g_{HH,k}\left(S_{HH}\alpha_{HV}\exp\left(j\phi_{HV}\right)+\alpha_{VV}\left(S_{VH}\exp\left(j\phi_{VV}\right)+S_{VV}\alpha_{VH}\exp\left(j\left(\phi_{VV}+\phi_{VH}\right)\right)\right)\right)\\ \;+I^{\prime }\cdot S_{VV}\alpha_{VV}^{2}\exp\left(2j\phi_{VV}\right)+w_{VH,k}\\ G_{HV,k}=g_{VV,k}\left(S_{HH}\alpha_{HV}\exp\left(j\phi_{HV}\right)+\alpha_{VV}\exp\left(j\phi_{VV}\right)\left(S_{HV}+\alpha_{VH}S_{VV}\exp\left(j\phi_{VH}\right)\right)\right)\\ \;+I^{\prime }\cdot S_{HH}+w_{HV,k}\\ G_{VV,k}=g_{VV,k}\alpha_{VV}\left(\alpha_{HV}\exp\left(j\left(\phi_{VV}+\phi_{HV}\right)\right)\left(S_{VH}+S_{HV}\right)+S_{VV}\alpha_{VV}\exp\left(2j\phi_{VV}\right)\right)\\ \;+I^{\prime }\cdot S_{VH}\alpha_{VV}\exp\left(j\phi_{VV}\right)+w_{VV,k} \end{array}\right.$$

Our goal is to estimate the PSM of moving target from the $G_{k}$ that includes the target echo and thermal noise of the receivers. As mentioned before, the variance of the thermal noise is assumed to be $\sigma_{w}^{2}$. Furthermore, the thermal noise is supposed to follow the Gaussian distribution. In [16], it has been proved that the noise components in the MF output, which are $w_{HH,k}$, $w_{VH,k}$, $w_{HV,k}$ and $w_{VV,k}$, follow the Gaussian distribution similarly and the variance is $T_{r}\sigma_{w}^{2}$. To obtain the real PSM of the target, three estimation methods are analyzed in the next Section.

## 3. PSM Estimation for the Moving Target

Instead of estimating the real PSM of the target, the PSM with relative amplitude and phase is estimated. With $S_{HH}$ as a reference, the relative PSM can be expressed as

(16) $$\bar{S}=\left[\begin{array}{c} \;1\;\frac{\left|S_{HV}\right|}{\left|S_{HH}\right|}\exp\left(j\phi_{HV}\right)\\ \frac{\left|S_{VH}\right|}{\left|S_{HH}\right|}\exp\left(j\phi_{VH}\right)\;\frac{\left|S_{VV}\right|}{\left|S_{HH}\right|}\exp\left(j\phi_{VV}\right) \end{array}\right]=\left[\begin{array}{c} \;1\;S_{HV/HH}\\ S_{VH/HH}\;S_{VV/HH} \end{array}\right]$$

where the $\phi_{VH}$, $\phi_{HV}$ and $\phi_{VV}$ are the phases of different polarized channels, and the $S_{VH/HH}$, $S_{HV/HH}$ and $S_{VV/HH}$ are the normalized scattering matrix parameters. To facilitate the following discussion, some notations are defined as follows

(17) $$\begin{array}{c} A_{HH}=S_{HH}+\alpha_{VH}\exp\left(j\phi_{VH}\right)\left(S_{VH}+S_{HV}\right)\\ A_{VH}=S_{HH}\alpha_{HV}\exp\left(j\phi_{HV}\right)+\alpha_{VV}\left(S_{VH}\exp\left(j\phi_{VV}\right)+S_{VV}\alpha_{VH}\exp\left(j\left(\phi_{VV}+\phi_{VH}\right)\right)\right)\\ A_{HV}=S_{HH}\alpha_{HV}\exp\left(j\phi_{HV}\right)+\alpha_{VV}\exp\left(j\phi_{VV}\right)\left(S_{HV}+\alpha_{VH}S_{VV}\exp\left(j\phi_{VH}\right)\right)\\ A_{VV}=\alpha_{VV}\left(\alpha_{HV}\exp\left(j\left(\phi_{VV}+\phi_{HV}\right)\right)\left(S_{VH}+S_{HV}\right)+S_{VV}\alpha_{VV}\exp\left(2j\phi_{VV}\right)\right) \end{array}$$

and

(18) $$\begin{array}{c} B_{HH}=I^{\prime }\cdot \alpha_{VV}S_{HV}\exp\left(j\phi_{VV}\right)\\ B_{VH}=I^{\prime }\cdot S_{VV}\alpha_{VV}^{2}\exp\left(2j\phi_{VV}\right)\\ B_{HV}=I^{\prime }\cdot S_{HH}\\ B_{VV}=I^{\prime }\cdot S_{VH}\alpha_{VV}\exp\left(j\phi_{VV}\right) \end{array}$$

Moreover, introducing the notation,

(19) $$x_{pq,k}=A_{c}\left(A_{pq}\exp\left(j\frac{4\pi v_{0}kT_{\mathrm{PRT}}}{\lambda }\right)+\frac{B_{pq}}{A}\exp\left(j\frac{4\pi d_{0}}{\lambda }\right)\right);p,q=H,V$$

where $A_{c}=A\exp\left(-j4\pi d_{0}/\lambda \right)$, $d_{0}$ is the target initial distance, $v_{0}$ is the radial velocity and $T_{\mathrm{PRT}}$ is the pulse repetition time. In the rest of the article, p,q are used to denoted H,V. Then Equation (15) can be rewritten as

(20) $$G_{pq,k}=x_{pq,k}+w_{pq,k}$$

For a static target, $x_{pq,1}=x_{pq,2}=\ldots =x_{pq,N}$. Using the PI method, $S_{pq}$ can be estimated by ${\hat{S}}_{pq}=\sum_{k=1}^{N}\left(G_{pq,k}/N\;\right)$, and the estimation of the relative PSM becomes

(21) $${\hat{S}}_{pq/HH\_\mathrm{PI}}=\frac{\sum_{k=1}^{N}G_{pq,k}}{\sum_{k=1}^{N}G_{HH,k}}$$

However, when the target moves, its echoes are incoherent. That means the motion of the target has impact on the measurements of the PSM. Generally, the velocity of the target can be estimated by the relative phase change of the echoes. In [17], the standard deviation of the estimation error is given as follows

(22) $$\sigma_{v}=\frac{\lambda }{2}\sqrt{6/\left[{\left(2\pi \right)}^{2}\chi {\left(NT_{\mathrm{PRT}}\right)}^{2}\right]}$$

where χ is the SNR of the input signal. With the estimated velocity ${\hat{v}}_{0}$, the PC method can be used to estimate the parameters of the scattering matrix by

(23) $${\hat{S}}_{pq}=\sum_{k=1}^{N}\left(\frac{G_{pq,k}}{A_{c}N}\cdot \exp\left(-j\frac{4\pi {\hat{v}}_{0}kT_{\mathrm{PRT}}}{\lambda }\right)\right)$$

Therefore, the estimation of the relative PSM is

(24) $${\hat{S}}_{pq/HH\_\mathrm{PC}}=\frac{\sum_{k=1}^{N}G_{pq,k}\exp\left(-j\frac{4\pi {\hat{v}}_{0}kT_{\mathrm{PRT}}}{\lambda }\right)}{\sum_{k=1}^{N}G_{HH,k}\exp\left(-j\frac{4\pi {\hat{v}}_{0}kT_{\mathrm{PRT}}}{\lambda }\right)}$$

It can be observed from Equations (21) and (24) that the PI method is a special case of the PC method. When the velocity of the target reduces to 0 m/s, PC has the same expression as PI. Additionally, for the PC method, accurate estimation of the PSM requires that the velocity of the target is estimated precisely. If the estimation error is large, the phase caused by the target’s motion cannot be compensated, leading to the inaccurate estimation of the PSM. Compared with the PC method, the estimation of the velocity is avoided in the MS method. The term $m_{pq,k}$ is added to indicate the selection state of the measurements

(25) $$m_{pq,k}=\left\{\begin{array}{c} 1,\;\mathrm{selected}\\ 0,\;\mathrm{unselected} \end{array}\right.,k=1,2,\ldots ,N$$

The integration results for different channels in MS method are

(26) $$G_{pq}=X_{pq}+W_{q}$$

where

(27) $$\begin{array}{c} G_{pq}=\sum_{k=1}^{N}m_{pq,k}G_{pq,k}\\ X_{pq}=\sum_{k=1}^{N}m_{pq,k}x_{pq,k}\\ W_{q}=\sum_{k=1}^{N}m_{pq,k}w_{pq,k} \end{array}$$

For arbitrary channel, the SNR of the integration terms is defined as:

(28) $${\mathrm{SNR}}_{pq}^{\mathrm{integration}}=\frac{E\left(X_{pq}X_{pq}^{*}\right)}{T_{r}\sigma_{w}^{2}\sum_{k=1}^{N}m_{pq,k}}=\frac{E\left(\sum_{k=1}^{N}m_{pq,k}x_{pq,k}\sum_{k=1}^{N}m_{pq,k}{\left(G_{pq,k}-w_{pq,k}\right)}^{*}\right)}{T_{r}\sigma_{w}^{2}\sum_{k=1}^{N}m_{pq,k}}$$

where $E\left(\cdot \right)$ represents the mathematical expectation. Since the thermal noise is assumed to follow the Gaussian distribution, and the mean and the variance are zero and $T_{r}\sigma_{w}^{2}$, respectively, Equation (28) can be simplified to

(29) $$\begin{array}{cc} {\mathrm{SNR}}_{pq}^{\mathrm{integration}} & =\frac{E\left(\sum_{k=1}^{N}m_{pq,k}x_{pq,k}\sum_{k=1}^{N}m_{pq,k}G_{pq,k}^{*}\right)}{T_{r}\sigma_{w}^{2}\sum_{k=1}^{N}m_{pq,k}}\\ & =\frac{E\left(\sum_{k=1}^{N}m_{pq,k}\left(G_{pq,k}-w_{pq,k}\right)\sum_{k=1}^{N}m_{pq,k}G_{pq,k}^{*}\right)}{T_{r}\sigma_{w}^{2}\sum_{k=1}^{N}m_{pq,k}}\\ & =\frac{E\left(\sum_{k=1}^{N}m_{pq,k}G_{pq,k}\sum_{k=1}^{N}m_{pq,k}G_{pq,k}^{*}-\sum_{k=1}^{N}m_{pq,k}w_{pq,k}{\sum_{k=1}^{N}m_{pq,k}\left(x_{pq,k}+w_{pq,k}\right)}^{*}\right)}{T_{r}\sigma_{w}^{2}\sum_{k=1}^{N}m_{pq,k}} \end{array}$$

Since for *N* observations, the measurement results are certain. Equation (29) can be further simplified to

(30) $$\begin{array}{cc} {\mathrm{SNR}}_{pq}^{\mathrm{integration}} & =\frac{{\left|G_{pq}\right|}^{2}-E\left(\sum_{k=1}^{N}m_{pq,k}w_{pq,k}\sum_{k=1}^{N}m_{pq,k}w_{pq,k}^{*}\right)-E\left(\sum_{k=1}^{N}m_{pq,k}w_{pq,k}\sum_{k=1}^{N}m_{pq,k}x_{pq,k}^{*}\right)}{T_{r}\sigma_{w}^{2}\sum_{k=1}^{N}m_{pq,k}}\\ & =\frac{{\left|G_{pq}\right|}^{2}}{T_{r}\sigma_{w}^{2}\sum_{k=1}^{N}m_{pq,k}}-1 \end{array}$$

It is clear that to improve the estimation performance, the SNR of the integration terms should be as high as possible. Besides, it should be pointed out that the term $m_{pq,k}$ cannot be all zero. When $m_{pq,1}=m_{pq,2}=\ldots =m_{pq,N}=0$, the term $G_{pq}=0$, and the SNR in (30) becomes meaningless. Another thing should be noticed is that to ensure the phase consistency of the selected measurements from different channels, the terms $m_{HH,k}$, $m_{VH,k}$, $m_{HV,k}$ and $m_{VV,k}$ should be equal, which means $m_{HH,1}=m_{VH,1}=m_{HV,1}=m_{VV,1},\ldots \ldots ,m_{HH,N}=m_{VH,N}=m_{HV,N}=m_{VV,N}$. Then the target term $X_{pq}$ can be estimated by the observation term $G_{pq}$ through minimizing the reciprocal of the first term of the SNR in Equation (30), and the criterion of the measurements selection can be expressed as

(31) $$\left\{\begin{array}{c} {\hat{X}}_{HH}=G_{HH};{\hat{X}}_{VH}=G_{VH};{\hat{X}}_{HV}=G_{HV};{\hat{X}}_{VV}=G_{VV}\\ \min_{m_{HH,k}}\;\left\{\frac{T_{r}\sigma_{w}^{2}\sum_{k=1}^{N}m_{HH,k}}{{\left|G_{HH}\right|}^{2}},\frac{T_{r}\sigma_{w}^{2}\sum_{k=1}^{N}m_{HH,k}}{{\left|G_{VH}\right|}^{2}},\frac{T_{r}\sigma_{w}^{2}\sum_{k=1}^{N}m_{HH,k}}{{\left|G_{HV}\right|}^{2}},\frac{T_{r}\sigma_{w}^{2}\sum_{k=1}^{N}m_{HH,k}}{{\left|G_{VV}\right|}^{2}}\right\} \end{array}\right.$$

The sequence of $m_{pq,1},m_{pq,2},\ldots ,m_{pq,N}$ has $2^{N}$ combinations. The combination, which makes the SNR in Equation (30) maximum, can be obtained by enumerating. For instance, for N=3, the value space of the sequence $m_{pq,1},m_{pq,2},\ldots ,m_{pq,N}$ is

(32) $$\left[\begin{array}{c} m_{pq,1}\\ m_{pq,2}\\ m_{pq,3} \end{array}\right]\in \left\{\left[\begin{array}{c} 1\\ 0\\ 0 \end{array}\right],\left[\begin{array}{c} 0\\ 1\\ 0 \end{array}\right],\left[\begin{array}{c} 0\\ 0\\ 1 \end{array}\right],\left[\begin{array}{c} 1\\ 1\\ 0 \end{array}\right],\left[\begin{array}{c} 0\\ 1\\ 1 \end{array}\right],\left[\begin{array}{c} 1\\ 0\\ 1 \end{array}\right],\left[\begin{array}{c} 1\\ 1\\ 1 \end{array}\right]\right\}$$

By substituting each element of the value space into Equation (30) and calculating the ${\mathrm{SNR}}_{pq}^{\mathrm{integration}}$ of each channel, then the combination which satisfies the criterion in Equation (31) can be obtained. With $x_{pq,k}=S_{pq/HH}x_{HH,k}$, the target integration results for the pq and HH channels satisfy

(33) $$X_{pq}=\sum_{k=1}^{N}m_{pq,k}x_{pq,k}=\sum_{k=1}^{N}m_{pq,k}S_{pq/HH}x_{HH,k}=S_{pq/HH}X_{HH}$$

Thus, the estimation of $S_{pq/HH}$ is

(34) $${\hat{S}}_{pq/HH\_\mathrm{MS}}=\frac{{\hat{X}}_{pq}}{{\hat{X}}_{HH}}=\frac{G_{pq}}{G_{HH}}$$

Based on Equation (34), the estimated target’s PSM is

(35) $$\hat{\bar{S}}=\left[\begin{array}{c} \;1\;\frac{G_{HV}}{G_{HH}}\\ \frac{G_{VH}}{G_{HH}}\;\frac{G_{VV}}{G_{HH}} \end{array}\right]$$

As is shown in Equation (35), no prior information are required in the MS method. Therefore, the PSM estimation of the target in non-uniform motion can also be solved. Performance of these three methods are analyzed in the next section.

## 4. Simulation Results and Discussion

In this section, the proposed method is evaluated using simulations. Subsequently, some metrics are defined herein. The polarized correlation coefficient (PCC) is selected to illustrate the performance of different methods. It can be defined as [18,19]

(36) $$PCC=\frac{\left|{S^{\prime }}^{H}\cdot \hat{S^{\prime }}\right|}{\sqrt{{S^{\prime }}^{H}S^{\prime }{\hat{S^{\prime }}}^{H}\hat{S^{\prime }}}}$$

where $S^{\prime }$ is the vector form of the relative PSM which is

(37) $$S^{\prime }={\left[1\;S_{VH/HH}\;S_{HV/HH}\;S_{VV/HH}\right]}^{T}$$

and ${\hat{S}}^{\prime }$ is the vector form of the estimated target’s PSM $\hat{\bar{S}}$. PCC indicates the similarity between two PSMs. In addition, based on Equations (19) and (20), the SNR of pq polarized channels can be defined as

(38) $${\mathrm{SNR}}_{pq}=\frac{{\left|A_{c}A_{pq}\right|}^{2}}{T_{r}\sigma_{w}^{2}}$$

and the average SNR of the four polarized channels is

(39) $$\mathrm{SN}R_{av}=\frac{\mathrm{SN}R_{HH}+\mathrm{SN}R_{VH}+\mathrm{SN}R_{HV}+\mathrm{SN}R_{VV}}{4}$$

### 4.1. PSM Estimation without System Errors

In this subsection, the system errors, including the cross-polarization isolation of the antennas, and the amplitude and phase difference of the channels, are supposed to be calibrated. Thus, the matrices R and T can be set to

(40) $$R=T=\left[\begin{array}{c} 1\;0\\ 0\;1 \end{array}\right]$$

Radar and target parameters are shown in Table 1. To analyze the performance of different methods, a point target was simulated with a ${\mathrm{SNR}}_{av}$ ranging from −10dB to 30dB. For each polarized channel, the peak of the target’s HRRP is used to compose the measured PSM. The velocity of the target can be obtained by adding a sinusoidal shift to the discrete time. The trajectories of the target in this paper do not represent the real, while they can be used to investigate the robustness of the method. The motion of the target in a particular Monte Carlo (MC) trial is

(41) $$v_{u}\left(k\right)=v_{u,0}+v_{u,0}^{2}\sin\left(\frac{\pi kT_{PRT}}{P}\right)$$

where *k* is the pulse number, the radial velocity $v_{u,0}$ is a uniform distribution on $\left[100\;m/s,150\;m/s\right]$, which means the Doppler frequency belongs to $\left[6.67\;\mathrm{kHz},10\;\mathrm{kHz}\right]$, *u* is the index of an arbitrary MC trial, and the control factor P=10. It can be observed from Equation (41) that when $P\gg \pi kT_{PRT}$ (e.g., P=500), the target’s motion becomes uniform. Besides, as this paper concentrates on the PSM estimation of moving targets, target detection is not discussed. With the simulated trajectories, measurements from a moving target can be obtained by the equations shown in Table 2, and the PSM estimation methods are carried out.

**Figure 2:**
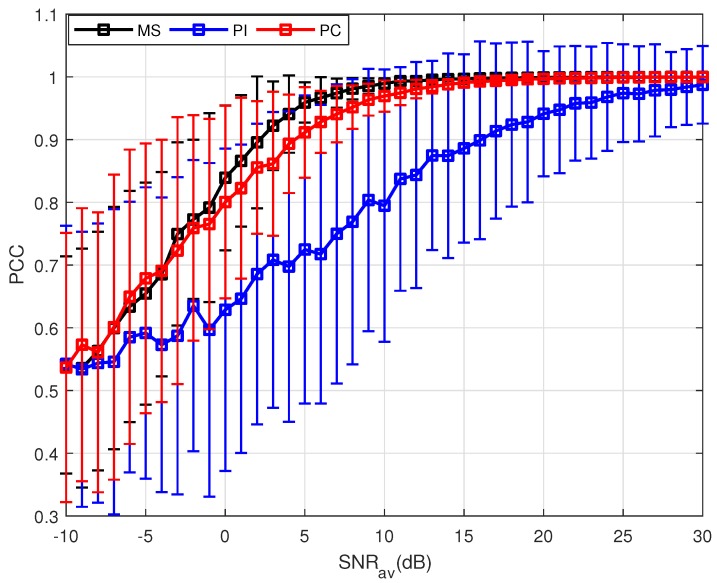
Results of three PSM estimation methods where P=500.

Utilizing the parameters shown in Table 1, 500 MC trials have been done at each SNR. The means and standard deviations (STD) of the PCC are plotted as curves and error bar, respectively. Figure 2 shows the performance of these three PSM estimation methods when the target experiences uniform motion. Since the target is not static, the echoes are not incoherent. Thus, direct sum of the echoes cannot obtain effective accumulation, leading to the worst performance of the PI method. For the PC and MS method, the PCC means increase to 1 with the increase of the ${\mathrm{SNR}}_{av}$, and the STD decreases owing to the same factor. As is mentioned before, the PC method uses the estimated velocity to compensate the phases of the measurements. However, it can be observed from Equation (22) that the accuracy of the velocity estimation is influenced by the SNR, and random errors in the estimation are inevitable. With a potentially inaccurate estimation, the phase generated by the target motion cannot be compensated completely, leading to the degradation in the PC results. By using partial measurements selected, the MS method gets the best performance. The essential reason is that by measurements selection, the echoes with similar phase are chosen, which means the selection coefficient $m_{pq,k}=1$. The phases of these selected measurements are approximatively coherent, leading to the best performance of the MS method.

**Figure 3:**
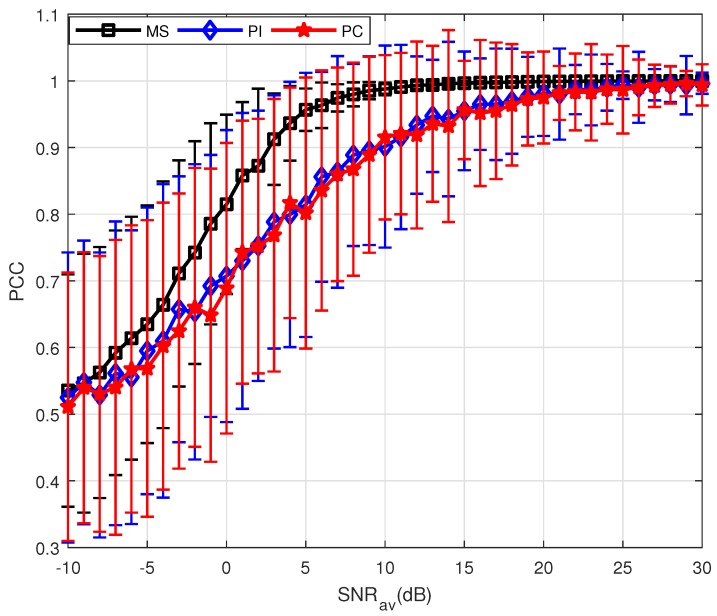
Results of three PSM estimation methods where P=10.

Furthermore, PSM estimation for a non-uniformly moving target is also analyzed. Figure 3 clearly demonstrates the performance of the PC method is highly affected by the target’s motion. For the non-uniform case, it is difficult to estimate the target velocity accurately, causing the incomplete compensation of the measurements. The performance of PC method significantly deteriorates compared with the results shown in Figure 2. Compared with PC, the MS method does not require any prior information about the target motion and thus it can be valid for the non-uniform motion.

**Figure 4:**
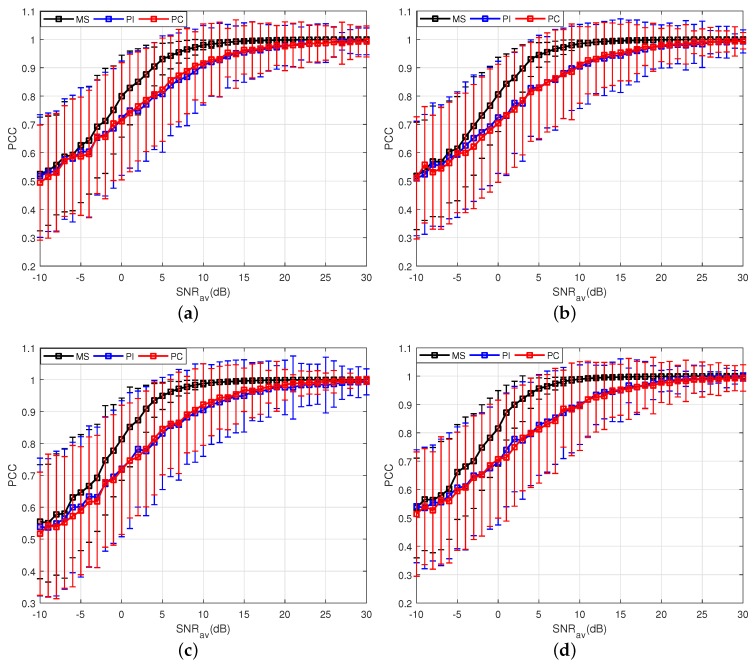
Results of three PSM estimation methods for different integrated pulses. (**a**) Results of three PSM estimation methods where *N* = 4; (**b**) Results of three PSM estimation methods where *N* = 6; (**c**) Results of three PSM estimation methods where *N* = 8; (**d**) Results of three PSM estimation methods where *N* = 10.

Besides, as mentioned in Section 2, in order to assure the constant target PSM, the CPI should be limited. Thus, the analysis of the proposed method for different integrated pulses *N* is given as follows. Here, the control factor is set to be P=10 and other parameters are same as those in Table 1. It can be observed from Figure 4 that the number of *N* has almost no influence on the properties of these three methods for estimating the moving target PSM. The proposed method still performs better than the other two methods when the CPI is short, and the performance of the MS method is not sensitive to the number of *N*. 

### 4.2. PSM Estimation with System Errors

In this subsection, the influence of the system errors on the PSM estimation is simulated. With the system errors of the well-known PARSAX radar system as a reference, the matrices are set as follows [20]

(42) $$R=T=\left[\begin{array}{c} \;1\;0.05\exp\left(j\frac{\pi }{180}\right)\\ 0.05\exp\left(j\frac{\pi }{180}\right)\;1.05\exp\left(j\frac{\pi }{180}\right) \end{array}\right]$$

**Figure 5:**
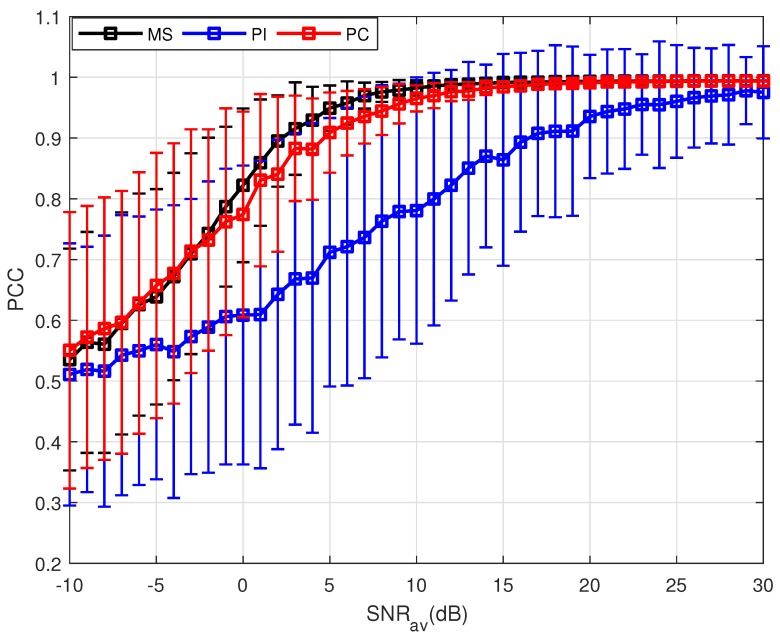
Results of three PSM estimation methods where P=500.

**Figure 6:**
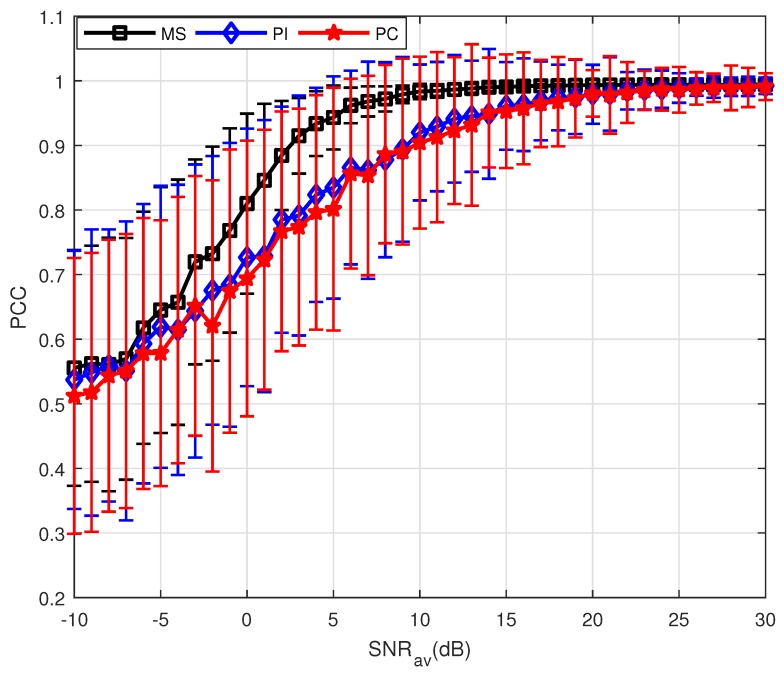
Results of three PSM estimation methods where P=10.

Other simulation parameters are same as those in Table 1. Similarly, the PSMs of targets with different motion states are estimated and the results are shown in Figure 5 and Figure 6. The performance of the MS method is the best among these three PSM estimation methods under two kinds of motion states, and the reason has been given in the last subsection. Here, the difference is that, compared with the results shown in Figure 2 and Figure 3, even if the SNR is high, the maximum value of the PCC mean is lower than 1. The reason is that when the isolation *I* of the transmitted is ignored, the limitation of Equation (34) is

(43) $$\lim_{\mathrm{SNR}\_av\to \infty }\;{\hat{S}}_{pq/HH\_\mathrm{MS}}=\lim_{\mathrm{SNR}\_av\to \infty }\;\frac{G_{pq}}{G_{HH}}=\frac{A_{pq}}{A_{HH}}$$

If the system errors do not exist, $A_{pq}$ is equal to $S_{pq}$ and the limitation of Equation (34) is $S_{pq/HH}$. However, in this subsection, the system errors are set as Equation (42), leading to the limitation is not equal to $S_{pq/HH}$. Therefore, the PCC mean is lower than 1.

## 5. Conclusions

For moving target PSM estimation, the measurement selection (MS) method is proposed in this paper. This method selects the measurements by the criterion based on the SNR of the integration echoes. Several numerical simulations and comparative analysis are conducted to demonstrate and validate the superior performance of the proposed method, especially when the target exhibits non-uniform motion, compared with the traditional methods. Furthermore, the authors plan to conduct research on the PSM estimation for the extended target.

## Figures and Tables

**Table 1:** Parameters for numerical experiments.

Component	Value	Component	Value
Carrier frequency	10 GHz	Target PSM	$\left[\begin{array}{c} \;1\;0.5j\\ 0.5j\;0.9 \end{array}\right]$
Mode	STSR	Control factor	P=500,10
Bandwidth	1 GHz	Initial position	5000 m
Pulse duration	100 μs	${\mathrm{SNR}}_{av}$	−10∼30dB
PRF	1000 Hz	Noise	Gaussian
Measurements	12	Trials of each SNR	500

**Table 2:** Methods for PSM Estimation.

	PI	PC	MS
HH	1	1	1
VH	$\frac{\sum_{k=1}^{N}G_{VH,k}}{\sum_{k=1}^{N}G_{HH,k}}$	$\frac{\sum_{k=1}^{N}G_{VH,k}\exp\left(-j\frac{4\pi {\hat{v}}_{0}kT_{\mathrm{PRT}}}{\lambda }\right)}{\sum_{k=1}^{N}G_{HH,k}\exp\left(-j\frac{4\pi {\hat{v}}_{0}kT_{\mathrm{PRT}}}{\lambda }\right)}$	$\frac{G_{VH}}{G_{HH}}$
HV	$\frac{\sum_{k=1}^{N}G_{HV,k}}{\sum_{k=1}^{N}G_{HH,k}}$	$\frac{\sum_{k=1}^{N}G_{HV,k}\exp\left(-j\frac{4\pi {\hat{v}}_{0}kT_{\mathrm{PRT}}}{\lambda }\right)}{\sum_{k=1}^{N}G_{HH,k}\exp\left(-j\frac{4\pi {\hat{v}}_{0}kT_{\mathrm{PRT}}}{\lambda }\right)}$	$\frac{G_{HV}}{G_{HH}}$
VV	$\frac{\sum_{k=1}^{N}G_{VV,k}}{\sum_{k=1}^{N}G_{HH,k}}$	$\frac{\sum_{k=1}^{N}G_{VV,k}\exp\left(-j\frac{4\pi {\hat{v}}_{0}kT_{\mathrm{PRT}}}{\lambda }\right)}{\sum_{k=1}^{N}G_{HH,k}\exp\left(-j\frac{4\pi {\hat{v}}_{0}kT_{\mathrm{PRT}}}{\lambda }\right)}$	$\frac{G_{VV}}{G_{HH}}$
